# Lipidomic profiling of extracellular vesicles derived from prostate and prostate cancer cell lines

**DOI:** 10.1186/s12944-018-0854-x

**Published:** 2018-09-08

**Authors:** Joshua S. Brzozowski, Helen Jankowski, Danielle R. Bond, Siobhan B. McCague, Benjamin R. Munro, Melanie J. Predebon, Christopher J. Scarlett, Kathryn A. Skelding, Judith Weidenhofer

**Affiliations:** 1 School of Biomedical Sciences and Pharmacy, University of Newcastle, Ourimbah, NSW Australia; 2 Cancer Research Program, Hunter Medical Research Institute, New Lambton Heights, NSW Australia; 3 School of Environmental and Life Sciences, University of Newcastle, Ourimbah, NSW Australia; 4 School of Biomedical Sciences and Pharmacy, University of Newcastle, Callaghan, NSW Australia

**Keywords:** Extracellular vesicles, Lipidomics, Prostate cancer

## Abstract

**Background:**

Extracellular vesicles (EVs) are produced and secreted from most cells of the body and can be recovered in biological fluids. Although there has been extensive characterisation of the protein and nucleic acid component of EVs, their lipidome has received little attention and may represent a unique and untapped source of biomarkers for prostate cancer diagnosis and prognosis.

**Methods:**

EVs were isolated from non-tumourigenic (RWPE1), tumourigenic (NB26) and metastatic (PC-3) prostate cell lines. Lipids were extracted and subsequently used for targeted lipidomics analysis for the quantitation of molecular lipid species.

**Results:**

A total of 187 molecular lipid species were quantitatively identified in EV samples showing differential abundance between RWPE1, NB26 and PC-3 EV samples. Fatty acids, glycerolipids and prenol lipids were more highly abundant in EVs from non-tumourigenic cells, whereas sterol lipids, sphingolipids and glycerophospholipids were more highly abundant in EVs from tumourigenic or metastatic cells.

**Conclusions:**

This study identified differences in the molecular lipid species of prostate cell-derived EVs, increasing our understanding of the changes that occur to the EV lipidome during prostate cancer progression. These differences highlight the importance of characterising the EV lipidome, which may lead to improved diagnostic and prognostic biomarkers for prostate cancer.

**Electronic supplementary material:**

The online version of this article (10.1186/s12944-018-0854-x) contains supplementary material, which is available to authorized users.

## Background

Cells release vesicles into the extracellular environment that include exosomes, microvesicles and apoptotic bodies, collectively termed extracellular vesicles (EVs). Exosomes (30–150 nm diameter) are formed via the endosomal pathway and released by fusion of a multivesicular body with the plasma membrane, whereas microvesicles (100–1000 nm) and apoptotic bodies (> 1000 nm) bud directly from the plasma membrane. EVs transport cargos of mRNAs, miRNAs, proteins, lipids and metabolites to target cells throughout the body, protecting them from degradation in the extracellular environment. Thus, EVs are involved in cell-cell communication, allowing for the activation of signalling pathways, and the transfer of cargo to affect cellular function [1]. Through this mechanism, EVs have been shown both in vivo and in vitro to promote tumour growth and metastasis in many cancers, including pancreatic [2, 3] and prostate cancers [4, 5]. However, EV-mediated communication is an important component of normal physiological function, with roles in priming the immune system and modulation of stem cell plasticity [6].

Due to the overlapping biophysical properties of the various EVs, analysis of a homogeneous population is difficult without the use of multiple isolation and purification steps, including differential ultracentrifugation, which are time consuming and are often still unable to yield a truly homogeneous population. Despite this limitation, EVs have received quite extensive characterisation of both their protein and nucleic acid (mRNA and miRNA) content. Indeed, there are now many proteins that show an enrichment in EV populations, including tetraspanins (CD9, CD63 and CD81), integrins, Tsg101 and Alix [7], with these proteins regularly used as markers of EV populations. However, one class of biomolecules, lipids, have been largely overlooked in studies characterising the composition of EVs.

Lipids have been implicated in multiple aspects of EV biogenesis and function. Due to the presence of lipid raft-associated proteins, including flotillin-1, in EVs, it is thought that lipid rafts may be influencing selective protein sorting into EVs [8]. Lipid rafts are areas of the plasma membrane that are rich in cholesterol and glycosphingolipids, acting as platforms for lipid raft-associated protein signalling. Further, the tetraspanins CD9 and CD81 are present in most EV preparations and both have known associations with cholesterol [9], suggesting that their specific incorporation and enrichment in EVs may be due to their associations with lipid rafts. Cholesterols, sphingomyelins and phosphatidylserine are the major components of lipid rafts, with all three lipids showing increased abundance in EVs when compared to their secreting cells [10–12].

Due to the unique architecture of the prostate and renal system, there is an increased interest in the use of urinary EVs for biomarker discovery in prostate cancer [13–15]. Prostatic secretions, which contain EVs, make their way into the urine and can be collected non-invasively and in large quantities compared to blood, making this technique ideal for assessment of prostate-specific vesicles and the identification of biomarkers for the diagnosis and prognosis of localised cancer [16, 17]. In addition to the potential of utilising EVs for diagnosis, their use as targeted therapies, and for the monitoring of pathological conditions, including cancer, has captured the attention of many researchers [18–20]. Clinical trials have already been performed, assessing the safety and efficacy of using exosomes as a therapeutic agent [21], however, there are many unanswered questions that remain pertaining to the complete biological composition of EVs. There is a lack in the understanding of the lipid composition of EVs and whether the lipid composition of EVs becomes altered during different pathological conditions. Further, it is not known how these alterations may influence, or be related to, the cargo recruitment and biological function of EVs. Herein, we have used a simple ultrafiltration approach for EV isolation, and targeted lipidomics to characterise the relative abundance of lipid species in three prostate cell line-derived EV populations. We have identified differences in the abundance of lipid species between non-tumourigenic, tumourigenic, and metastatic prostate cell line-derived EVs, highlighting the potential importance of the EV lipidome to cancer progression and diagnosis.

## Methods

### Cell culture

Non-tumourigenic prostate epithelial RWPE1 (CRL-11609), their chemically transformed, tumourigenic derivative WPE1-NB26 (CRL-2852), and prostate bone metastasis PC-3 (CRL-1435) cell lines were purchased from the American Type Culture Collection (ATCC; Cryosite, South Granville, NSW, Australia) and used within seven (RWPE1 and PC-3) and twelve (WPE1-NB26) passages. RWPE1 and WPE1-NB26 (herein referred to as NB26) cells were maintained in Keratinocyte Serum-Free Media (KSFM; Gibco, Thermo Fisher Scientific, North Ryde, NSW, Australia) containing the supplied growth supplements. PC-3 cells were maintained in Ham’s F-12 K (Kaighn’s Modification) Medium (Gibco) supplemented with 10% foetal bovine serum (FBS; Bovogen Biologicals, Interpath, Heidelberg West, VIC, Australia). Cells were maintained in a humidified incubator at 37 °C with 5% CO_2_.

### EV isolation and nanoparticle tracking analysis

Cells were grown to approximately 50% confluency in T175 flasks before media was aspirated and cells washed twice in sterile phosphate buffered saline (PBS; Thermo Fisher). KSFM, without growth supplements, was added to RWPE1 and NB26 cells. Serum-free Ham’s F-12 K was added to PC-3 cells. Cells were incubated in a humidified incubator at 37 °C with 5% CO_2_ for 48 h before media was collected for EV isolation.

EVs were collected using a modified ultrafiltration protocol, as previously described [22]. Briefly, 150 mL of media per collection was centrifuged for 20 min, 4 °C, 2000 × g to pellet cells and cellular debris. Clarified media was then sequentially passed through a 0.22 μm polyethersulfone (PES) syringe filter (Merck Millipore, Bayswater, VIC, Australia) and a 0.1 μm PES vacuum filter unit (Nalgene, Thermo Fisher). The 0.1 μm filtered media was then concentrated with an Amicon 100,000 MWCO Ultra-15 centrifugal filter unit (Merck Millipore) at 4 °C, 4000 × g. Flowthrough was discarded and centrifugation was repeated until the entire sample had been processed. Retentates were washed twice with 0.1 μm filtered PBS and collected into Protein LoBind tubes (Eppendorf, North Ryde, NSW, Australia) and stored at − 80 °C until use.

EV size and concentration was determined as previously described [22] using Nanoparticle Tracking Analysis (NTA) with a NanoSight NS300 and NTA v3.1 software (Malvern, ATA Scientific, Taren Point, NSW, Australia). Briefly, samples were diluted in 0.1 μm-filtered PBS and illuminated using a 405 nm (violet) laser. A scientific CMOS camera recorded 3 × 60s videos for each sample. NTA software was used to analyse the Brownian motion of particles in the captured videos and to generate analysis reports.

### Western blot

Western blotting was performed as previously described [22], with protein equivalent to 1 × 10^8^ EVs per lane (as determined using NTA). Briefly, EVs were added to sample loading buffer with or without reducing agents, boiled, and run on 4–12% Bis-Tris gels (Novex, Thermo Fisher). After transferring to nitrocellulose, blots were blocked with 5% skimmed milk powder in Tris buffered saline with 0.1% Tween-20 (TBST). Non-reduced blots were probed with 4 μg/mL mouse anti-CD9 [1AA2] (a gift from C. Prof Leonie Ashman, University of Newcastle, NSW, Australia), 1:1000 mouse anti-CD63 (BioVision, California, USA) and 1:1000 mouse anti-CD29/ITGB1 [MEM-101A] (EXBIO, Vestec, Czech Republic) primary antibodies diluted in TBST. Reduced blots were probed with 1:500 mouse anti-Alix (Santa-Cruz, Texas, USA) primary antibody diluted in 1% skim milk powder in TBST. Primary antibodies were detected with goat anti-mouse, horseradish peroxidase-conjugated secondary antibody (Bio-Rad, Gladesville, NSW, Australia). Proteins were detected using enhanced chemiluminescence (ECL) and images captured using an Amersham Imager 600 (GE Healthcare, Rydalmere, NSW, Australia).

### Lipid extraction

Six biological replicates of EVs from each cell line were collected and batch processed to minimise variation in lipid extraction between samples. Prior to lipid extraction, a total of 1 × 10^10^ EVs per sample were pelleted at 18,000 rpm, 3 h, 4 °C using a Hettich 1195-A fixed angle rotor in a Hettich Micro 220R refrigerated centrifuge. The Centrifugation Parameters Calculator, developed by the Laboratory of Molecular Human Genetics, Research Institute of Physical-Chemical Medicine, Moscow, Russia (http://vesicles.niifhm.ru [23]) was used to calculate the pelleting time, using a vesicle density of 1.08 g/cm^3^, and a complete sedimentation “cut-off” size of 50 nm diameter.

Lipids were extracted from EVs using a modified Folch extraction protocol [24]. Briefly, EV pellets were resuspended in an ice cold chloroform:methanol mix (2:1) with 10 mg/L of internal standards PC(19:0/19:0) and PG(17:0/17:0) (Avanti Polar Lipids, Alabama, USA). Samples were vortexed and then mixed at 950 rpm, 20 min, 22 °C with a Thermomixer C (Eppendorf). Samples were centrifuged at 15,000 rpm for 10 min at room temperature and the supernatant collected into a LoBind tube. Samples were completely dried using a centrifugal evaporator and stored at − 80 °C.

### MS analyses

Extracted lipids were processed and detected by Metabolomics Australia (Bio21 Institute, Melbourne, VIC, Australia) as previously described [25]. Liquid chromatography electrospray ionisation-tandem mass spectrometry (LC ESI-MS/MS) was performed using an Agilent 1290 LC system and an Agilent 6490 triple quadrupole mass spectrometer (Agilent Technologies, Mulgrave, VIC, Australia). Equal volumes of each EV lipid sample were combined to create a pooled quality control (QC) sample. An injection volume of 5 μL was used for EV lipids, with QC samples run after every five EV samples. LC separation was performed using ZORBAX eclipse plus C18 column (2.1 × 100 mm, 1.8 μm) (Agilent) maintained at 60 °C. Mobile phase solutions were water, acetonitrile and isopropanol (5:3:2 *v*/v for A and 1:9:90 v/v for B), both with 10 mM ammonium formate. Columns were run using a gradient of A and B solutions over 18 min at a flow rate of 0.4 mL/min. Metabolites were detected in positive ionisation mode using multiple reactions monitoring.

### Data processing

The MS data were processed with Agilent Mass Hunter software. The resultant raw data, indicative of relative abundance (AUC) was further processed using Microsoft Excel for Mac v16.12 and filtered to remove metabolites with a CV > 20%. Missing values were imputed, and the raw data Log_2_ transformed and normalised to the QC samples using MetaboAnalyst 4.0 [26], a web-based tool for the comprehensive analysis of metabolomics data. MetaboAnalyst was further used for principal component analysis (PCA) and the generation of heatmaps. Pie charts were created using the base functions in RStudio (RStudio, MA, USA), and the Metabolomics R package used to create boxplots of metabolites.

### Lipid nomenclature

Lipids are abbreviated as follows: sterol lipids – cholesteryl ester (CE), oxidised cholesteryl ester (oxCE); sphingolipids – ceramide (Cer), dihydroceramide (DHCer), ganglioside GM3 (GM3), hexa-ceramides with differing glycan chains (Hex1Cer, Hex2Cer, Hex3Cer), sphingomyelin (SM); glycerolipids – diacylglyceride (DG), triacylglyceride (TG); glycerophospholipids – phosphatidylcholine (PC), phosphatidylethanolamine (PE), phosphatidylglycerol (PG), phosphatidylinositol (PI), phosphatidylserine (PS) and the lyso (L) species (LPC and LPE). Alkyl ether and plasmalogen linkages are denoted by O- and P- respectively.

Lipids are named according to the LIPID MAPS classification system ‘Headgroup(*sn*1/*sn*2)’, where *sn* refers to the radyl side-chains [27, 28]. The side-chain structures are denoted as carbon chain length:number of double bonds and are provided for each chain where they could be determined, or as a total number of all carbons and double bonds where individual chains could not be determined.

### Statistics

To determine differences between individual lipid species between EV samples, the Log_2_ transformed data were analysed using an unpaired t-test, correcting for comparisons using the Benjamini and Hochberg false discovery rate with a q value of 5% (analyses available as Additional file 1). Data were considered statistically significant when q ≤ 0.05 with *p* values reported. These statistical analyses were performed using GraphPad Prism v7.0d (GraphPad Software, CA, USA).

## Results

**Fig. 1:**
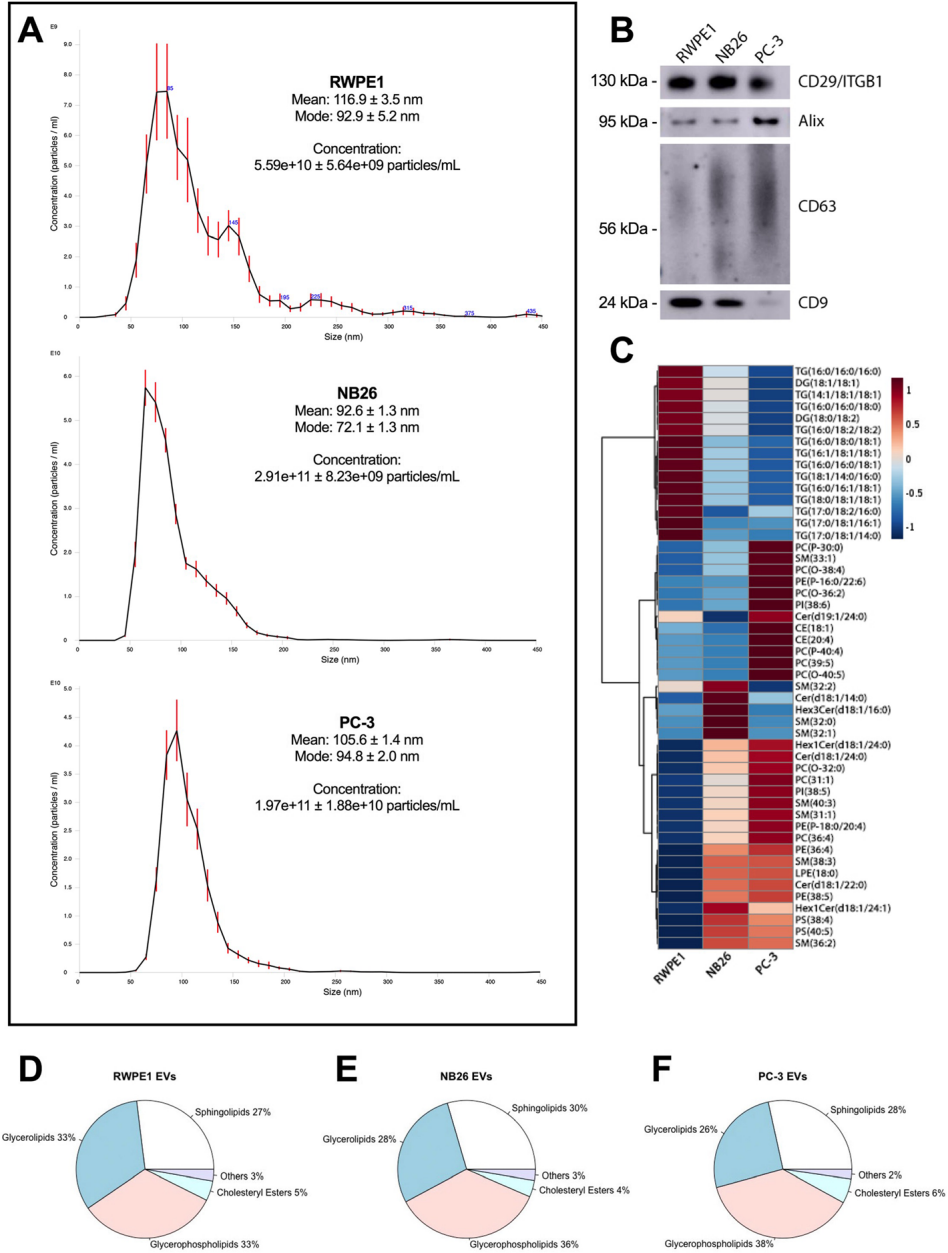
EVs from tumourigenic cell lines have differences in molecular lipid composition compared to EVs from non-tumourigenic cells. **a** NTA characterisation of EVs size distribution and concentration. **b** Detection of common EV-enriched proteins CD29, Alix, CD63 and CD9 by western blot using 1 × 10^8^ EVs per lane. **c** Heatmap of distinct clusters of enriched lipid species in RWPE1, NB26 and PC-3 EVs generated using the Euclidian distance measure and Ward clustering in MetaboAnalyst. **d-f** The overall composition of RWPE1, NB26 and PC-3 EVs based on the abundance of lipids in each lipid category. Sphingolipids included Cer, DHCer, GM3, HexCer and SM species, glycerolipids included DG and TG species, glycerophospholipids included LPC, LPE, PC, PE, PI, PG and PS species, others included acylcarnitine and ubiquinone

EVs isolated from non-tumourigenic (RWPE1), tumourigenic (NB26) and metastatic (PC-3) prostate cell lines had mean and mode sizes in the range of typically defined exosomes, as assessed by NTA (Fig. 1a). RWPE1 EVs had the lowest overall concentration compared to NB26 and PC-3 EVs, with NB26 having the highest overall concentration, in accordance with previous reports [29, 30]. All EVs were positive for the common EV markers CD29/ITGB1, Alix, CD63 and CD9 by western blot (Fig. 1b), however PC-3 EVs had minimal CD9 expression in comparison to RWPE1 and NB26 EVs, which is expected as CD9 expression is lower in PC-3 cells compared to RWPE1 [31].

### Characterisation of EV lipids

Targeted lipidomics was performed on EVs, using six replicate collections each, allowing for the quantitation of 187 lipid species using a log-transformed, QC normalised relative abundance that included lipids from the fatty acid, sterol lipid, sphingolipid, glycerolipid and glycerophospholipid lipid categories. Principal component analysis was performed on the dataset showing clear separation between the cell lines and clustering of the samples within the cell lines (see Additional file 2). The relative composition of EVs in the identified lipid classes differed slightly between RWPE1, NB26 and PC-3 cell lines (Fig. 1d-f; Table 1), where a decrease in abundance of glycerolipids, and an increase in abundance of sphingolipids and glycerophospholipids was observed in NB26 and PC-3 EVs compared to RWPE1. Further, MetaboAnalyst was used to generate a heatmap, using the Euclidean distance measure and Ward clustering, displaying the top 50 features as determined by t-test, highlighting the enrichment and clustering of various lipid species in EV groups (Fig. 1c).

**Table 1:** Mean abundance of lipid categories between RWPE1, NB26 and PC-3 cell-derived EVs

Lipid Category	RWPE1	NB26	PC-3
Fatty Acids	17.400	15.710	13.800
Sterol Lipids	9.696	8.741	12.050
Sphingolipids	11.690	12.800	12.660
Glycerolipids	14.470	12.770	11.930
Glycerophospholipids	10.200	10.890	11.970
Prenol Lipids	11.520	10.310	9.832

Abundance was measured as Log-transformed, normalised AUC

### The abundance of lipid species varies between EVs

#### Glycerolipids

**Fig. 2:**
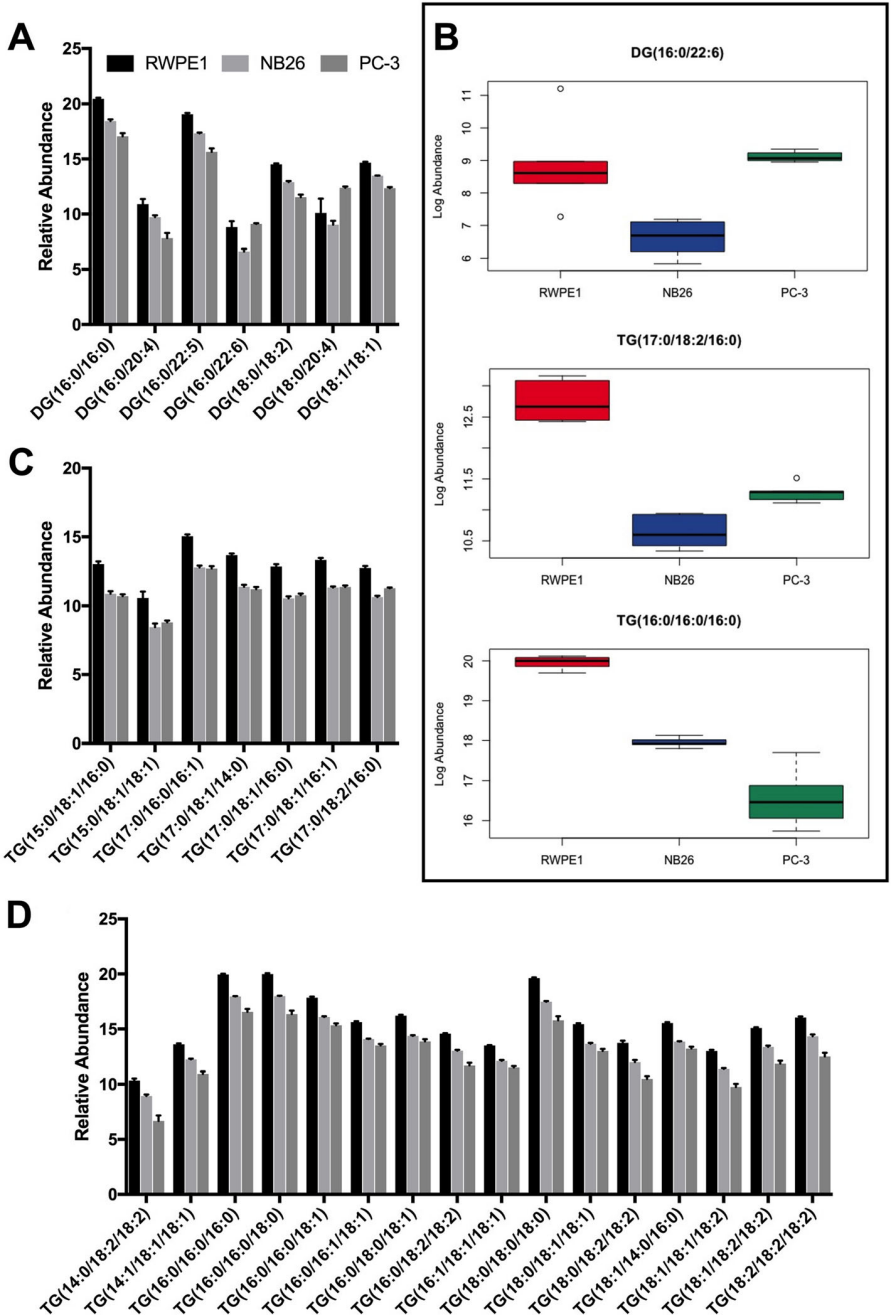
EVs from tumourigenic cells show lower abundance of glycerolipid species compared to EVs from non-tumourigenic cells. **a** Selected DG species showing a decrease in relative abundance of all glycerolipid species in NB26 and PC-3 EVs compared to RWPE1, except DG(18:0/20:4), which had higher abundance in PC-3 EVs. **b** Box plots of the representative glycerolipids DG(16:0/22:6), TG(17:0/18:2/16:0) and TG(16:0/16:0/16:0) showing the relative abundance of each lipid species in EVs. **c** TG species with a C15 or C17 acyl chain length at the sn1 position showed similar or increased abundance in PC-3 EVs compared to NB26 EVs. **d** TG species with a C14, C16 or C18 acyl chain length at the sn1 position had decreased abundance in NB26 and PC-3 compared to RWPE1 EVs

The abundance of both diacylglycerol (DG) and triacylglycerol (TG) species was decreased in NB26 and PC-3 EVs compared to RWPE1 EVs. For DG species combined, there were significant decreases in abundance between RWPE1 and NB26 EVs (*p* < 0.0001), RWPE1 and PC-3 EVs (*p* < 0.0001) and NB26 and PC-3 EVs (*p* = 0.0037) (Table 2). The only exception was observed in DG (18:0/20:4) for which PC-3 EVs had a + 2.28 Log_2_ fold change (FC) increased abundance compared to RWPE1 EVs and a + 3.35 FC increased abundance compared to NB26 EVs (Fig. 2a). Further, DG (16:0/22:6) abundance was significantly decreased in NB26 EVs with a − 2.21 FC compared to RWPE1 EVs and a − 2.49 FC compared to PC-3 EVs (Fig. 2b). As there were for DG species, there were decreases in the overall abundance of TG species between RWPE1 and NB26 EVs (*p* < 0.0001), RWPE1 and PC-3 EVs (*p* < 0.0001) and NB26 and PC-3 EVs (*p* = 0.0002) (Table 2). Of the detected TG species, there was a larger proportion containing polyunsaturated fatty acids (PUFA) compared to monounsaturated fatty acids (MUFA) or saturated fatty acids (SFA). The three detected SFA species TG (16:0/16:0/16:0) (Fig. 2b), TG (16:0/16:0/18:0) and TG (18:0/18:0/18:0) had the highest overall relative abundance in all EV samples (Fig. 2d), possibly suggesting that SFA TG species are important for the unique curvature of EV membranes. Interestingly, PC-3 EVs, when compared to NB26 EVs, had consistently lower abundance of all TG species with an even number of total carbons in the fatty acid (FA) chains (i.e. those with either C14, C16 or C18 in the *sn*1 position). However, in TG species with a total odd number of FA carbons (i.e. those with a C15 or C17 in the *sn*1 position) there was similar abundance between PC-3 and NB26 EVs (Fig. 2b-c), with one of these species, TG (17:0/18:2/16:0) having a significant + 0.64 FC increase in PC-3 EVs compared to NB26 EVs.

**Table 2:** Mean abundance of lipid classes in RWPE1, NB26 and PC-3 EVs

Lipid Category	RWPE1	NB26	PC-3
Lipid Class			
Fatty Acids			
Acylcarnitine	17.400	15.710	13.800
Sterol Lipids			
CE	8.859	7.755	12.300
oxCE	10.520	8.105	9.854
Free Cholesterol	12.800	15.770	14.230
Sphingolipids			
Cer	10.350	11.070	11.000
DHCer	13.170	12.780	12.350
GM3	12.170	10.280	8.758
Hexa-Cer	8.252	12.660	10.900
SM	13.230	14.310	14.740
Glycerolipids			
DG	14.000	12.530	11.860
TG	14.590	12.850	11.960
Glycerophospholipids			
LPC	8.435	9.050	9.028
LPC-O	11.070	9.540	10.930
LPC-P	7.026	6.213	8.378
LPE	8.583	11.140	11.240
LPE-P	9.712	10.680	10.430
PC	11.320	12.610	14.590
PC-O	10.210	11.310	14.700
PC-P	11.300	11.120	13.950
PE	8.178	11.540	11.000
PE-O	8.637	8.757	9.221
PE-P	10.070	11.360	11.330
PG	11.560	9.773	7.798
PI	8.754	9.905	11.970
PS	7.748	13.190	11.580
Prenol Lipids			
Ubiquinone	11.520	10.310	9.832

Abundance was measured as Log-transformed, normalised AUC

#### Glycerophospholipids

**Fig. 3:**
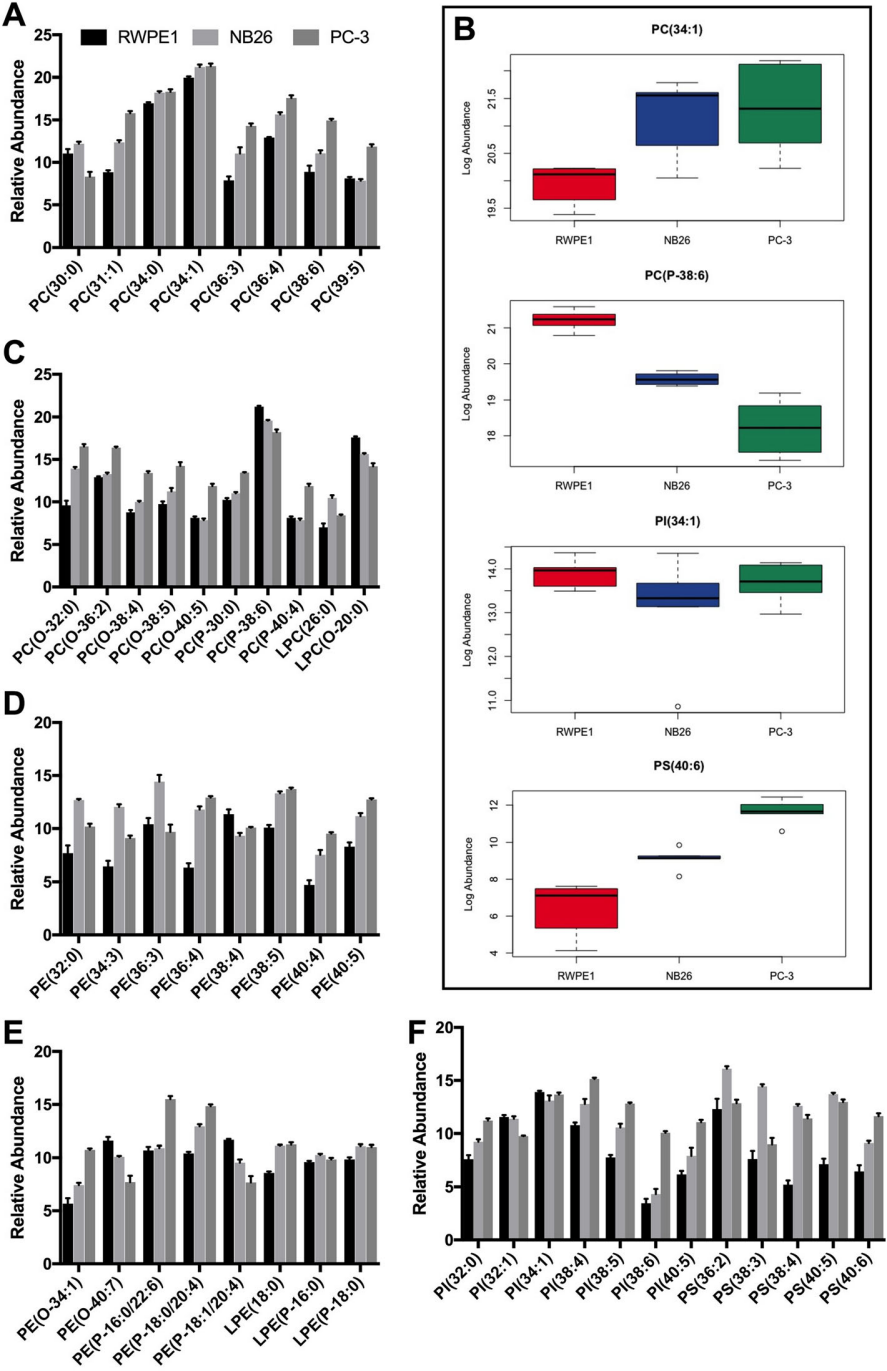
EVs from metastatic cells show higher abundance of glycerophospholipid species. **a** PC species showing increased relative abundance in PC-3 EVs compared to RWPE1 and NB26 EVs. PC(30:0) shows highest abundance in NB26 EVs with PC-3 EVs having the lowest abundance of this species. **b** Box plots of the representative glycerophospholipids PC(34:1), PC(P-38:6), PI(34:1) and PS(40:6) showing their relative abundance in EVs. **c** The relative abundance of alkyl (O-), alkenyl (P-) and Lyso (L) PC species showing an overall enrichment in PC-3 EVs. **d** The relative abundance of PE species in EVs. NB26 EVs had the highest abundance of PE species overall. **e** The relative abundance of alkyl, alkenyl and Lyso PE species in EVs. **f** The relative abundance of PI and PS species in EVs. PC-3 EVs had the highest overall abundance of PI species, whereas NB26 EVs had the highest overall abundance of PS species

NB26 and PC-3 EVs were enriched in glycerophospholipids compared to RWPE1 EVs, with glycerophosphocholine (PC) and glycerophosphoethanolamine (PE) species accounting for the highest proportion of glycerophospholipid abundance in all samples. Of the PC species, PC (34:0), PC (34:1) (Fig. 3a-b) and PC (P-38:6) (Fig. 3c) showed the highest abundance. RWPE1 EVs had a significantly higher abundance of PC (P-38:6), with a + 1.64 FC compared to NB26 EVs and a + 3.00 FC compared to PC-3 EVs (Fig. 3b). Overall, the abundance of ether-linked PC species was significantly higher in PC-3 EVs, with a + 3.76 FC increase compared to RWPE1 EVs (*p* < 0.0001) and a + 3.17 FC increase compared to NB26 EVs (*p* < 0.0001) (Table 2). For PE species, NB26 and PC-3 EVs had an overall higher relative abundance compared to RWPE1 EVs (Fig. 3d), however for ether-linked PE species (Fig. 3e), RWPE1 EVs showed a significant increase in abundance with a + 0.59 FC increase compared to NB26 EVs (*p* = 0.0021) and a + 0.55 FC increase compared to PC-3 EVs (*p* = 0.0034) (Table 2). PC-3 EVs had the highest relative abundance of glycerophosphoinisitol (PI) species detected with only PI (34:1) showing no significant differences in abundance between samples (Fig. 3b/f). Glycerophosphoserine (PS) species were most abundant in NB26 EVs with the exception of PS (40:6) which showed highest abundance in PC-3 EVs (Fig. 3b/f; Table 2).

#### Sphingolipids, fatty acids and sterol lipids

**Fig. 4:**
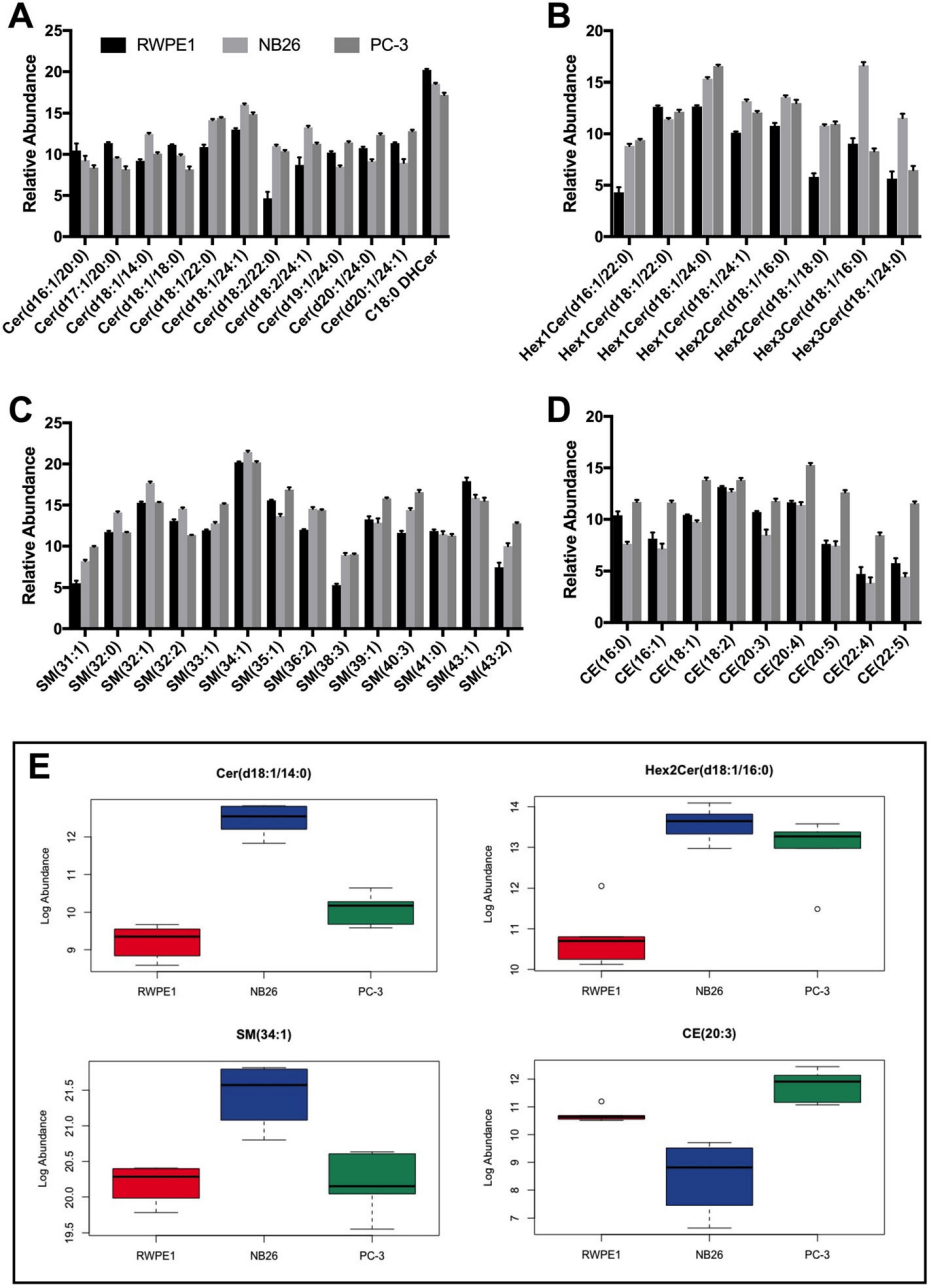
EVs from tumourigenic cells show higher abundance of sphingolipid and sterol lipid species. **a** Most Cer species showed an overall increased abundance in NB26 and PC-3 EVs compared to RWPE1 EVs. However, several individual Cer and DHCer species including Cer(d16:1/20:0) and C18:0 DHCer had increased abundance in RWPE1 EVs. **b** Hexa-Cer species were more highly abundant in NB26 and PC-3 EVs, with both Hex3Cer species showing specific enrichment in NB26 EVs. **c** NB26 and PC-3 EVs had the highest overall abundance of SM species, with RWPE1 EVs only showing highest abundance in SM(43:1). **d** PC-3 EVs had the highest overall abundance of CE species. **e** Box plots of the representative sphingolipid and sterol lipid species Cer(d18:1/14:0), Hex2Cer(d18:1/16:0), SM(34:1) and CE (20:3) in EVs

Ceramide (Cer) and sphingomyelin (SM) species were the most enriched classes of sphingolipids detected in EVs. There were 17 Cer species identified, accounting for 30.1% of RWPE1, 28.8% of NB26 and 29.0% of PC-3 EV sphingolipid composition. Further, there were significant decreases in total Cer abundance in RWPE1 EVs with a − 0.72 FC decrease compared to NB26 EVs (*p* = 0.0004) and a − 0.65 FC decrease compared to PC-3 EVs (*p* = 0.0010) (Table 2). RWPE1 EVs were significantly enriched in Cer (d17:1/20:0), Cer (d18:1/18:0) and dihydroceramide (DHCer) C18:0 compared to both NB26 and PC-3 EVs (Fig. 4a). NB26 EVs were significantly enriched in Cer(d18:1/14:0) (Fig. 4e) and Cer (d18:2/24:1) compared to both RWPE1 and PC-3 EVs.

Several Hexa-ceramide (HexCer) species were identified, including four Hex1Cer, two Hex2Cer and two Hex3Cer species (Fig. 4b/e). For Hex1Cer species, NB26 EVs had a + 2.82 FC increase (*p* < 0.0001) and PC-3 EVs had a + 3.22 FC increase (*p* < 0.0001) in overall abundance compared to RWPE1 EVs, with no significant difference between NB26 and PC-3 EVs. However, Hex1Cer(d18:1/22:0) was significantly increased in RWPE1 EVs with a + 1.20 FC increase compared to NB26 (*p* = 0.0002) and a + 0.50 FC increase compared to PC-3 EVs (*p* = 0.0171). Overall, Hex2Cer species had a + 4.54 FC increase in NB26 EVs (*p* < 0.0001) and a + 4.33 FC increase in PC-3 EVs (*p* < 0.0001) compared to RWPE1 EVs, with no significant difference between NB26 and PC-3 EVs. Hex3Cer species were only significantly increased in abundance in NB26 EVs with a + 7.37 FC increase compared to RWPE1 EVs and a + 7.53 FC increase compared to PC-3 EVs (*p* < 0.0001). However, there was no significant difference between RWPE1 and PC-3 EVs for Hex3Cer species abundance. There were 22 SM species identified, accounting for 49.8% of RWPE1, 48.2% of NB26 and 50.3% of PC-3 EV sphingolipid composition with RWPE1 EVs having a significant decrease in total SM abundance, with a − 1.08 FC decrease compared to NB26 EVs (*p* < 0.0001) and a − 1.51 FC decrease compared to PC-3 EVs (*p* < 0.0001) (Table 2). The most highly abundant SM species in all EVs was SM (34:1) (Fig. 4c/e). Several SM species displayed differential abundance across all EV samples, with RWPE1 EVs having the highest abundance of SM(43:1), NB26 EVs having the highest abundance of SM(32:0) and SM(32:2) and PC-3 EVs having the highest abundance of SM (39:1) and SM (43:2) to name a few (Fig. 4c).

There were two acylcarnitine species identified, acylcarnitines C17:0 and C18:1. Acylcarnitine C17:0 showed a − 1.73 FC decrease in NB26 EVs compared to RWPE1 EVs and a further − 1.86 FC decrease in PC-3 EVs compared to NB26 EVs (see Additional file 1). For acylcarnitine C18:1, there was a − 1.66 FC decrease in NB26 EVs compared to RWPE1 EVs and a further − 1.95 FC decrease in PC-3 EVs compared to NB26 EVs (see Additional file 1). Additionally, nine cholesteryl ester (CE) species were detected (Fig. 4d), with PC-3 EVs having the highest overall abundance and NB26 EVs having the lowest overall abundance (Table 2). For all CE species, PC-3 EVs showed a significantly higher abundance compared to both RWPE1 and NB26 EVs. Further, RWPE1 EVs had a significant increase in abundance of three CE species compared to NB26 EVs with a + 2.77 FC in CE (16:0), a + 0.66 FC in CE (18:1) and a + 2.21 FC in CE (20:3) (Fig. 4e).

## Discussion

Although there is increasing interest in the use of EVs for both diagnostic and therapeutic potential in a variety of pathologies, the understanding of the complete physical composition of vesicles from different tissue and cell types is still lacking. Many studies have focussed on the protein and nucleic acid component of EVs, however, understanding the lipid component of EVs from various cell and disease types represents an important, yet missing component. The transfer of biologically active lipids and lipid metabolites has emerged as a mechanism used by cancer cells to alter energy pathways within the tumour microenvironment. Many tumours, including prostate, show increased uptake and de novo synthesis of cholesterols and cholesteryl esters [32, 33], as well as the accumulation of triacylglycerols as energy reservoirs [34]. As EVs are known to transfer lipids and lipid-related proteins to affect target cell function [35–37], we employed a targeted lipidomics approach on our prostate cell-derived EVs, allowing for the specific identification and quantitation of lipid species.

We detected relatively high numbers of DG, TG and CE species. It has been previously reported that the detection of large numbers of these species in EVs is due to the presence of contaminating lipoproteins and/or lipid droplets in EV isolates [12]. We utilised ultrafiltration over the traditional ultracentrifugation method for the isolation of EVs, as it has higher yields of vesicles reported [38]. Further, ultrafiltration is better suited for translation into a clinical setting due to the relative speed of the protocol, the larger volumes of sample able to be processed and the fact that no specialist equipment is required [39]. Ultrafiltration is more likely to co-isolate other particles, resulting in a heterogeneous mixture, and as such the DG, TG and CE species detected here may have arisen from lipid droplets. However, far from being considered a confounding factor, the detection of these species may have clinical relevance in terms of diagnosis of disease.

The accumulation of CEs in prostate cancer is associated with the progression and metastasis of the tumour, with this CE accumulation being identified in lipid droplets within prostate cancer cells [40], suggesting that the co-isolation of lipid droplets with EVs may be beneficial for prostate cancer diagnosis. Further, it has recently been shown that CEs, specifically CE (18:1) (cholesteryl oleate), can discriminate between prostate cancer, non-tumour, and benign prostatic hyperplasia [40, 41]. Although we have shown that CEs were higher in abundance in metastatic PC-3 EVs compared to the normal RWPE1 EVs we saw a decrease in CE abundance in NB26 EVs compared to RWPE1 EVs, however, this may be a result of the lineage of the NB26 cell line. The NB26 cell line is a derivative of the RWPE1 cell line, formed by exposure of RWPE1 cells to *N*-methyl-*N*-nitrosourea and two generations of injection and growth in nude mice [42]. Due to this lineage, and although the NB26 cell line has a tumourigenic and invasive phenotype, it may retain many similarities to the RWPE1 cell line. In addition to the potential diagnostic value of CE detection in EV isolates, it has also been demonstrated that the metastatic prostate cancer cell line DU145 accumulates unsaturated TG species when undergoing the epithelial to mesenchymal transition [34]. Therefore, the detection of these lipid species, whether they be from EVs, lipoproteins or lipid droplets, may provide important information relating to the progression and prognosis of prostate cancer.

An increase in ether-linked lipids has been shown to cause higher numbers of EVs (exosomes) to be secreted from cells. PC-3 cells treated with an ether lipid precursor, hexadecylglycerol, resulted in increased levels of ether-linked lipids in both cells and their secreted exosomes, and alterations to the protein composition of the exosomes [43]. Here, we observed a large fold change increase in the abundance of ether-linked PC species in PC-3 EVs compared to RWPE1 and NB26 EVs, with NB26 and PC-3 also having the highest concentration of EVs isolated. Increased PC abundance has been identified in breast cancer, with higher levels identified in cancerous tissue compared to healthy tissue [44]. PC species have also been associated with poor prognosis, and inclusion of a PC species in a biomarker panel consisting of the lipids Cer (d18:1/24:1), SM (d18:2/16:0) and PC (16:0/16:0) had prognostic significance and this lipid signature could identify patients with poor prognosis [45]. In accordance with this three-lipid signature, the present study identified Cer (d18:1/24:1) in all samples, with lower abundance in RWPE1 (non-tumourigenic) and higher abundance in NB26 and PC-3 (tumourigenic, metastatic) EVs, suggesting a role for this ceramide in the progression of prostate cancer. As we were unable to determine the individual acyl chains of SM and PC species, it is unknown whether SM (d18:2/16:0) or PC (16:0/16:0) were detected in our EV samples.

A large proportion of the sphingolipids identified in this study belonged to the Cer and SM classes. Sphingolipids, including ceramides have been implicated in the production and release of EVs, however there is conflicting evidence regarding the role of ceramide in this process. Using the oligodendroglial precursor cell line, Oli-neu, treated with three unrelated sphingomyelinase inhibitors to inhibit the formation of ceramide from SM, it was found that there was a reduction in the number of exosomes released [46]. Conversely, in PC-3 cells treated with a glucosylceramide synthase inhibitor, there was no change in the number of exosomes released [47]. Additionally, acylcarnitines are utilised by cancer cells for energy production where they are shuttled from the cell membrane to the mitochondria to undergo fatty acid oxidation for downstream use in the TCA cycle. Although there were only two acylcarnitine species detected in this study, we saw that there was a significant stepwise decrease in abundance in EVs from non-tumourigenic, tumourigenic and metastatic prostate cell lines. In a prospective study of prostate cancer, an association between plasma acylcarnitine concentration and cancer progression was seen [48]. Acylcarnitine concentration was shown to decrease in patients with high grade prostate cancer and increase in patients with more advanced prostate cancer or who had succumbed to their disease. Although the associations were statistically significant using conventional statistics of linearity, when correcting for multiple comparisons using the false discovery rate, the associations were no longer significant. Similar findings have been seen in hepatocellular carcinoma where there was an increase in the accumulation of acylcarnitines with long FA chains (>C14) during disease progression [49]. As we saw a decrease in the concentration of long chain acylcarnitines in our EVs, this may suggest that there is a higher cellular utilisation of these metabolites that restrict their abundance in EVs.

Together, and as identified by heatmap analysis, the differences in abundance of lipid species identified between EVs from non-tumourigenic, tumourigenic and metastatic prostate cells highlights that these differences may be a source of biomarkers for prostate cancer diagnosis and prognosis. Further, the known functional roles of particular lipid species in cancer cells suggests that, in addition to providing encapsulation for EV cargo, the vesicle membrane composition along with the EV cargo may possibly have important roles in driving cancer progression.

## Conclusions

In summary, we have identified differences in the abundance of the molecular lipid species of EVs from non-tumourigenic, tumourigenic and metastatic prostate cells in vitro. These differences highlight the potential for the use of EV-derived lipids for diagnostic and prognostic evaluation of prostate cancer. This work increases our understanding of the biological makeup of EVs and may pave the way for the development of a lipid signature biomarker for different disease states. By understanding the molecular lipid composition of EVs, we may better understand the roles of the EV lipidome in cell signalling events.

## Additional files

Additional file 1: BH Corrected Multiple Comparisons of EV Lipid Species. Lipid species data were analysed using an unpaired t-test, correcting for multiple comparisons using the Benjamini and Hochberg FDR with a q value of 5%. The workbook contains 3 sheets, one for each comparison: RWPE1 v NB26, RWPE1 v PC-3 and NB26 v PC-3. (XLSX 57 kb)

Additional file 2: Principal Components Analysis (PCA) plots of EV sample groups. PCA was performed on samples using MetaboAnalyst. The cell line groups were clearly separated from each other, whilst the samples within each cell line group were tightly clustered. (DOCX 54 kb)

## Abbreviations

ANOVA: Analysis of variance
EV: Extracellular vesicle
FA: Fatty acid
FBS: Foetal bovine serum
KSFM: Keratinocyte Serum-Free Media
MUFA: Monounsaturated fatty acid
NTA: Nanoparticle tracking analysis
PBS: Phosphate buffered saline
PUFA: Polyunsaturated fatty acid
QC: Quality control
SFA: Saturated fatty acid
