# Supplementary material for: Lipidomic profiling of extracellular vesicles derived from prostate and prostate cancer cell lines
Source: Lipids Health Dis. 2018 Sep 8;17:211. doi: 10.1186/s12944-018-0854-x (PMC6128989; doi:10.1186/s12944-018-0854-x)
Supplement: Supplementary file 2 — Principal Components Analysis (PCA) plots of EV sample groups. PCA was performed on samples using MetaboAnalyst. The cell line groups were clearly separated from each other, whilst the samples within each cell line group were tightly clustered. (DOCX 54 kb) [file 12944_2018_854_MOESM2_ESM.docx]

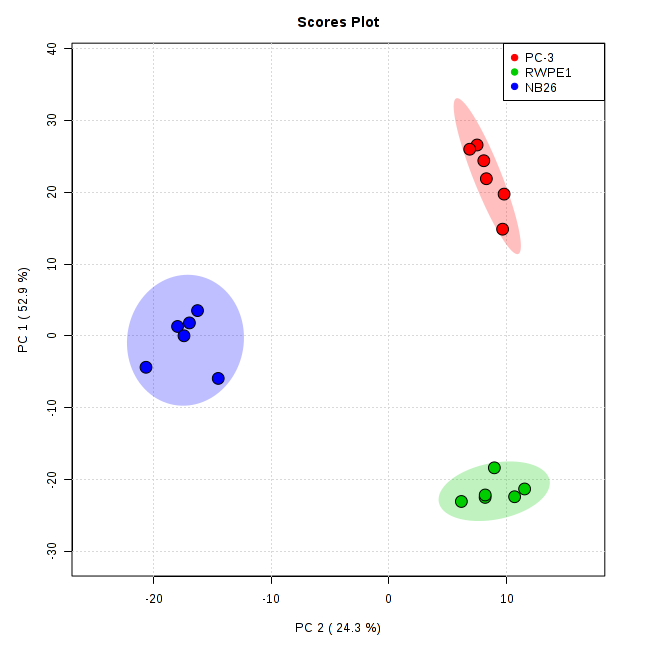


Additional File 2. Principal Components Analysis (PCA) plots of EV sample groups. MetaboAnalyst was used for the generation of the PCA plot using the log-transformed, normalised data. The cell line groups were clearly separated from each other, whilst the samples within each cell line group were tightly clustered.
